# A Web-Based Decision Aid (myAID) to Enhance Quality of Life, Empowerment, Decision Making, and Disease Control for Patients With Ulcerative Colitis: Protocol for a Cluster Randomized Controlled Trial

**DOI:** 10.2196/15994

**Published:** 2020-07-10

**Authors:** Andrew H Kim, Afaf Girgis, Neda Karimi, Alexandra J Sechi, Joseph Descallar, Jane M Andrews, Corey A Siegel, Susan J Connor

**Affiliations:** 1 Ingham Institute for Applied Medical Research South Western Sydney Clinical School The University of New South Wales Sydney Australia; 2 Department of Gastroenterology Liverpool Hospital Sydney Australia; 3 IBD Service Department of Gastroenterology & Hepatology Royal Adelaide Hospital Adelaide Australia; 4 Faculty of Medicine University of Adelaide Adelaide Australia; 5 Section of Gastroenterology and Hepatology Dartmouth-Hitchcock Medical Center Lebanon, NH United States

**Keywords:** shared decision making, decision aid, ulcerative colitis

## Abstract

**Background:**

Patients with ulcerative colitis (UC) often face complex treatment decisions. Although shared decision making (SDM) is considered important, tools to facilitate this are currently lacking for UC. A recent pilot study of a novel Web-based decision aid (DA), my Actively Informed Decision (myAID), has suggested its acceptability and feasibility for informing treatment decisions and facilitating SDM in clinical practice.

**Objective:**

This paper describes the study protocol of the myAID study to assess the clinical impact of systematic implementation of myAID in routine UC management.

**Methods:**

The myAID study is a multicenter, cluster randomized controlled trial (CRCT) involving 22 Australian sites that will assess the clinical efficacy of routine use of myAID (intervention) against usual care without access to myAID (control) for UC patients. Participating sites (clusters) will be randomly allocated in a 1:1 ratio between the 2 arms. Patients making a new treatment decision beyond 5-aminosalicylate agents will be eligible to participate. Patients allocated to the intervention arm will view myAID at the time of recruitment and have free access to it throughout the study period. The effect of the myAID intervention will be assessed using the results of serial Web-based questionnaires and fecal calprotectin at baseline, 2 months, 6 months, and 12 months. A Web-based questionnaire within 2-4 weeks of referral will determine early change in quality of decision making and anxiety (both arms) and intervention acceptability (intervention arm only).

**Results:**

Study recruitment and funding began in October 2016, and recruitment will continue through 2020, for a minimum of 300 study participants at baseline at the current projection. The primary outcome will be health-related quality of life (Assessment of Quality of Life-8D), and secondary outcomes will include patient empowerment, quality of decision making, anxiety, work productivity and activity impairment, and disease activity. In addition, we aim to determine the predictors of UC treatment decisions and outcomes and the cost-effectiveness of implementing myAID in routine practice. Feedback obtained about myAID will be used to determine areas for improvement and barriers to its implementation. Completion of data collection and publication of study results are anticipated in 2021.

**Conclusions:**

myAID is a novel Web-based DA designed to facilitate SDM in UC management. The results of this CRCT will contribute new evidence to the literature in comparing outcomes between patients who routinely access such decision support intervention versus those who do not, across multiple large inflammatory bowel disease centers as well as community-based private practices in Australia.

**Trial Registration:**

Australian New Zealand Clinical Trial Registry ACTRN12617001246370 http://anzctr.org.au/Trial/ Registration/TrialReview.aspx?ACTRN=12617001246370

**International Registered Report Identifier (IRRID):**

DERR1-10.2196/15994

## Introduction

### Background

Ulcerative colitis (UC) is a chronic disabling inflammatory bowel disease (IBD). It affects about 40,000 people in Australia [1], with its incidence and burden rising globally [2]. Although half of these patients may be managed with 5-aminosalicylates (5-ASAs) alone [3], the rest will face decisions about other treatments that require long-term immunosuppression and even colectomy. These decisions are complex because they involve not only the identification of the best treatment strategy to prevent symptoms and progression of the disease but also consideration of the trade-offs of the available treatment options that differ in their efficacy and potential risks, modes of delivery, and dosing intervals. Therefore, patients’ values and preferences heavily influence treatment choice and adherence, and a more collaborative and empowering approach to help navigate the complex benefit-risk profiles of these treatment options is important in guiding the decision-making process [4,5].

A recent survey confirmed that such a process of patient engagement or shared decision making (SDM) is desired by patients with UC [6]. SDM helps the doctor and the patient to collaborate on management decisions, which can lead to improved quality of life (QoL) and likelihood of achieving health goals, while lowering the demand for health care resources and improving patients’ health care experience [7]. Many health care organizations have embraced SDM as an important part of health care standards and patient-centered care [8-10].

Decision aids (DAs) are tools developed to facilitate SDM by presenting patients with evidence-based information in a patient-friendly format and encouraging active engagement in the decision-making process. A recent Cochrane review indicates that their use in other chronic diseases can improve patient knowledge and reduce decisional conflict and the number of patients remaining undecided or being passive in the decision-making process [11]. With increasing acceptance and familiarity of online tools, Web-based DAs are gaining popularity [6,12,13].

Although using DAs use has the potential to facilitate SDM and participatory medicine in UC management, their uptake and application in clinical practice to date have been limited. Available electronic health technologies have suffered from high attrition rates [14], and patient and clinician perspectives on the best approach to the use of these tools in routine clinical practice remain poorly understood. A participatory health research design to increase involvement of patients and clinicians in their development and subsequent implementation has been suggested as a potential way of increasing their effectiveness and overcoming these limitations.

Recently, a new Web-based DA was developed for use in UC, which, for the first time, includes an interactive video discussing both medical and surgical treatment options in UC management, with the aim of facilitating SDM. Patients and a multidisciplinary panel of clinical experts were involved in its design, with a rigorous evaluation process [15] and close reference to the International Patient Decision Aids Standards checklist [16]. Originally developed in the United States, the DA was modified for use by an Australian audience in this study and named myAID (for my Actively Informed Decision; Emmi Solutions). A pilot study confirmed the acceptability of myAID to both patients and clinicians as a feasible SDM tool for UC management (Kim AH et al, unpublished data, 2020). To investigate whether routine use of myAID to promote SDM will translate to improvement of meaningful outcomes for patients, we designed a national cluster randomized controlled trial (CRCT) to test its efficacy against usual care. The CRCT is hereafter referred to as *the myAID study*.

### Research Objectives

The overall aim of this study is to assess the impact of systematic use of myAID by eligible patients with UC on patient-reported UC clinical outcomes over a period of up to 12 months. Specific objectives of this study are as follows:

1. Comparison of changes in health-related QoL (primary outcome) from baseline to 6-month follow-up in patients accessing myAID (intervention arm) versus patients receiving usual care.
2. Comparison of differences in patient empowerment, quality of decision making, anxiety, work productivity, and disease activity including steroid use, hospital visits, and colectomy (secondary outcomes) in patients accessing myAID versus patients receiving usual care at the 6-month follow-up.
3. Determine the predictors of health literacy in patients with UC and the relationship between health literacy, decisional conflict, and treatment choices.
4. Determine the cost-effectiveness of implementing myAID in routine practice.
5. Investigate the views of study participants about myAID and determine areas for improvement or barriers to implementation in clinical practice.

## Methods

### Trial Design

**Figure 1:**
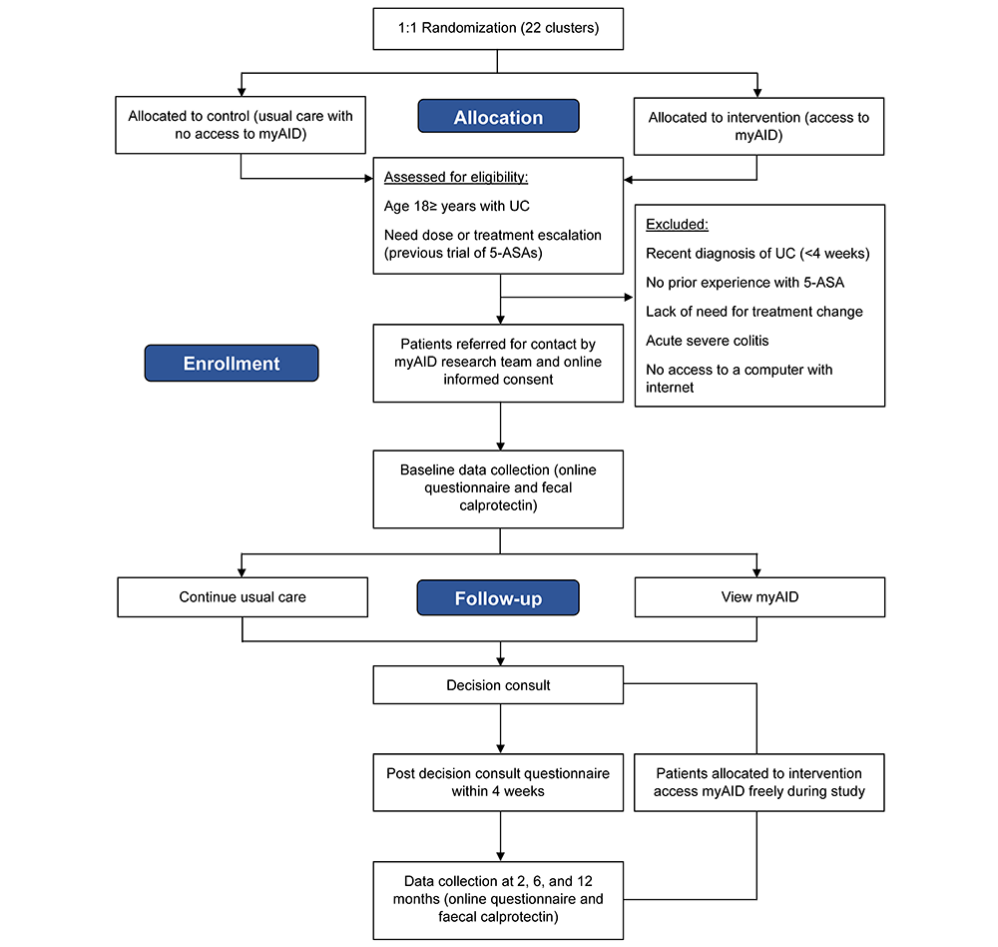
myAID study flowchart. 5-ASA: 5-aminosalicylates; UC: ulcerative colitis; myAID: my Actively Informed Decision. *Decision consult, if arranged, will be arranged within 2-4 weeks of referral. **Post decision consult (if arranged) or follow-up questionnaire to be completed within 2-4 weeks of referral.

The myAID study is a multicenter CRCT (see Figure 1) using a parallel arm design. Sites will be randomly allocated in a 1:1 ratio between intervention (routine use of myAID) and control (usual care without access to myAID) arms, with each site constituting a single cluster. Patients managed privately by the clinicians practicing at that site will also be grouped into the same cluster. As the intervention requires viewing and interacting with myAID, this is an open-label study with participants and their treating clinician and team being aware of their group allocation. Although only the patients, their family, and clinicians allocated to the intervention arm will be able to access and view myAID during the course of the study, participants from both groups may access other resources either sought independently or as recommended by their treating team, such as printed or online resources via patient organization websites. The use of such resources will be documented via participant questionnaires.

### Setting

This study will be undertaken at 22 sites in 5 of the 6 states in Australia, including large public IBD clinics and community-based private practices in urban and regional areas. Patient enrollment commenced in October 2016, with 21 of the 22 sites currently enrolling participants. All data collection (all participants) and viewing of myAID (intervention participants) will be undertaken off-site.

### Study Population and Eligibility

#### Trial Inclusion Criteria

Patients presenting to the outpatient clinic at the participating sites will be eligible if they (1) have a diagnosis of UC, (2) are 18 years or older, (3) need to make a new decision about their UC management following a lack of response to (previous or current) 5-ASA treatment, and (4) can consent and read the myAID information online in English.

New decisions about management will have to specifically involve discussions around dose escalation, addition or change in treatment. This may include 5-ASA dose increase or addition of either oral and topical treatment, addition or dose increase of steroids, thiopurines, methotrexate, tofacitinib, and biologic agents as well as discussion regarding better treatment adherence. Consented patients could continue as study participants if they underwent colectomy after study enrollment, provided that this decision was made after baseline assessment and viewing of myAID (for the intervention group).

#### Trial Exclusion Criteria

The exclusion criteria will be as follows:

1. New diagnosis of UC (diagnosis within 4 weeks before referral) or no prior experience with 5-ASA
2. Lack of need for treatment escalation, addition or change in treatment (including patients who have already undergone colectomy at the time of referral),
3. Current episode of acute severe colitis requiring inpatient treatment
4. Not having access to a computer with internet outside of the clinic.

### Study Procedure

#### Randomization

Given the cluster randomization design, all patients are allocated to either the intervention or control arm depending on the allocation of their referring site. When this project was conceptualized, block randomization was used to randomly allocate the original 14 invited sites equally into the intervention and control groups. Since the beginning of the study, 8 additional sites have joined recruitment and were allocated in pairs to the intervention or control group using predetermined block randomization, stratified by size and practice type. The block randomization was developed by the team statistician (JD), and the master copy, which informs the group allocation of site pairs, is held by the only investigator who is not directly involved in any study-related contact (eg, recruitment) or clinical care of participants (AG). Sites were informed of their allocation only after study approval by their local ethics governance.

#### Intervention—my Actively Informed Decision

**Figure 2:**
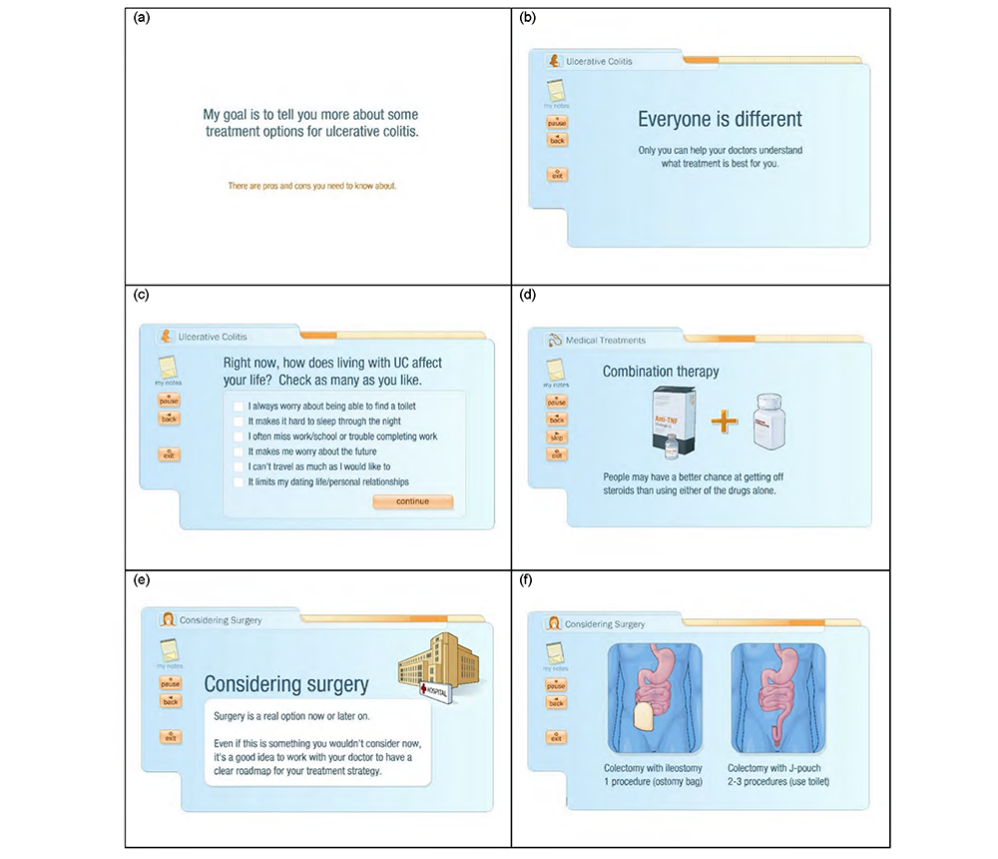
Screenshots of myAID (myAID@2015 Emmi Solutions, LLC). myAID: my Actively Informed Decision.

The development, key features, and feasibility testing of the myAID intervention were previously described in a pilot study (Kim AH et al, unpublished data, 2020). In brief, myAID (Emmi Solutions) is a Web-based multimedia DA incorporating an interactive video (Figure 2), which has been designed to help prepare viewers for decision making about their UC treatment. Its content has been structured to deliver information aimed at improving the viewer’s understanding of UC as well as the available medical and surgical treatments, including their potential benefits and risks, and to elicit the viewer’s treatment goals and preferences through a series of interactive questions. The current version is accessible using a computer with an internet connection but is not downloadable or accessible on a mobile device. It takes approximately 32 min to view uninterrupted, with the actual time varying with content watched and time taken with interactive elements.

Patients (and their invited family members) allocated to the intervention arm will have free access to myAID during the period of the study through their designated URL, which will be provided to them upon completion of their baseline Web-based questionnaire. They will be able to view myAID at any location using a computer, provided there is internet access. All patients will confirm their first complete viewing of myAID via their Web-based questionnaire. Patients will report any technical issues with regard to access or use of myAID via email for assistance by the research team. myAID access by these study participants will also be tracked by Emmi Solutions and reported back to the myAID study group on a monthly basis. The treating clinicians allocated to the intervention arm will similarly have free access to myAID during the study period.

#### Recruitment and Informed Consent

Patients who are eligible for the study will be identified by their respective consultant gastroenterologist who will obtain verbal consent (or written consent, if specifically required by their local ethics governance) for the interested patient’s contact details to be forwarded onto the myAID study group. Referred patients will then receive a study invitation email with an attached electronic participant information booklet, followed by a phone call from the study research assistant to confirm receipt of the study information and provide a verbal explanation of the study. A chance to win an Aus $200 (US $123) gift voucher will be used as an incentive for participation and completion of questionnaires. Those who wish to proceed will then be sent a further email with their unique study ID and a URL, which they will use to provide online consent and complete the baseline questionnaire and a laboratory request form for fecal calprotectin (FC) testing within 3 weeks from the time of the referral to the study. Patients who did not comply with the required timeframe can be re-referred provided they still meet the study inclusion criteria.

#### Study Flow and Data Collection

All patients completing their baseline Web-based questionnaire will be entered into the study database and continue to be followed up by the myAID study group for 12 months unless they withdraw their consent. All participating sites will continue to provide usual care, although we will ask that a brief follow-up, either in the form of a face-to-face or telephone consultation (referred to as a *decision consult*), be arranged at 2-4 weeks after patient referral to the study if possible. Although not mandated for the study, this was felt to be important by clinicians in our pilot study to facilitate SDM.

Web-based questionnaires (using the SurveyMonkey platform) are administered to study participants at baseline, 2-4 weeks (or following the *decision consult* if arranged), 2 months, 6 months (primary and secondary outcome assessment), and 12 months (long-term impact). Table 1 summarizes the outcome measures captured at each timepoint. Study participants will be sent a URL for the questionnaires via email at each of these time points, as this was deemed acceptable and feasible from the pilot study (Kim AH et al, unpublished data, 2020).

All participants will be asked to submit their stool samples at baseline, 2 months, 6 months, and 12 months. Each site will provide the study participant with a preassembled stool sampling kit at the time of referral. Study participants will be asked to submit their samples to their local Sonic Healthcare pathology laboratory (nationwide pathology provider owned by Sonic Healthcare Limited), where the FC will be measured using a quantitative fluoroenzyoimmunoassay (EliA Calprotectin 2; Thermo Fisher). Participants residing outside of Adelaide in South Australia will use the SA Pathology laboratory (statewide pathology provider for the public health sector in South Australia) that uses the same assay for FC testing. Participants who do not have access to either of these labs will be asked to submit their samples to an alternative local pathology lab. All FC measurements will be performed according to the manufacturer’s instructions without knowledge of patient data. Concentrations will be expressed as micrograms per gram of stool.

We will accept patient referrals until 2020 to reach our target recruitment sample and follow-up participants for up to 12 months, thereby completing data collection in 2021.

**Table 1:** myAID study outcome measures.

Outcome measures	Baseline (within 3 weeks of referral)	Within 4 weeks	2 months	6 months	12 months
Assessment of Quality of Life-8D	✓^a^	N/A^b^	✓	✓	✓
Health Education Impact Questionnaire	✓	N/A	✓	✓	✓
Health Literacy Questionnaire	✓	N/A	✓	✓	✓
Decisional Conflict Scale	✓	✓	✓	✓	✓
Trust in Physician Scale	✓	✓	✓	✓	✓
Hospital Anxiety and Depression Scale-Anxiety	✓	✓	✓	✓	✓
Simple Clinical Colitis Activity Index	✓	N/A	✓	✓	✓
Fecal calprotectin	✓	N/A	✓	✓	✓
Clinical outcomes	✓	N/A	✓	✓	✓
Work productivity and activity index	✓	N/A	✓	✓	✓
myAID acceptability	N/A	✓	N/A	N/A	N/A

^a^✓: included.

^b^N/A: not applicable.

#### Measures

##### Patient Demographics, Disease, and Treatment Characteristics

Upon consenting, all patients will complete a Web-based questionnaire to provide the following:

1. Socioeconomic data—age, gender, ethnicity, postal code, language spoken at home, relationship, education, employment and smoking status, and health insurance coverage.
2. Clinical data—time since diagnosis, disease extent, previous hospital visits and admissions for UC, comorbidities, UC-related surgical history, previous and current treatment history including systemic corticosteroid use, main setting for clinical care (public vs private), and access to an IBD nurse.

#### Review

##### Outcome Measures

The primary outcome for the study is *QoL* as measured by the 35-item Assessment of Quality of Life-8D (AQoL-8D) [17]. AQoL-8D is a health-related multi-attribute utility QoL instrument that measures 8 dimensions within 2 *super-dimensions*: Physical (independent living, pain, and senses) and psychosocial (mental health, happiness, coping, relationships, and self-worth). AQoL-8D has previously been used in economic evaluation studies in both Crohn disease (CD) [18] and UC [19]. Each item is scored on a 4- to 6-point Likert scale based on a recall period of 7 days, and these are reduced to a single utility score using an algorithm that also generates an index number for each of the 8 dimensions and for the 2 *super-dimensions*.

The secondary outcomes for the study are as follows:

1. *Empowerment*. This will be measured by the Health Education Impact Questionnaire 3.0 (heiQ) [20] using 4 of its dimensions (32 items): positive and active engagement in life, constructive attitudes and approaches, self-monitoring and insight, and emotional well-being. Items are recorded on a 4-point Likert scale from 1 to 4, where 1=strongly disagree and 4=strongly agree. The items in each dimension are summed, and the sum is divided by the number of items to generate a score for each dimension. The heiQ was developed to assess the outcomes of patient education programs, with higher scores indicating better self-management and knowledge, except for the emotional well-being scale, which is reversed.
2. *Health literacy*. This will be measured by the 44-item Health Literacy Questionnaire (HLQ) [21]. The HLQ covers 9 domains, each consisting of 4 to 6 items measured on either 4- or 5-point Likert scales. There is no one overall summative score; the score for each domain is generated by following a computerized algorithm using SPSS or Microsoft Excel. The included domains capture how the patients engage, access, and use health information and services and provide an opportunity for reflection of the quality of health and social service provision.
3. *Quality of decision making*. This will be measured by the 16-item Decisional Conflict Scale (DCS) [22] and the 11-item Trust in Physician Scale (TPS) [23]. DCS measures uncertainty in making a choice, modifiable factors contributing to the uncertainty, and perceived effective decision making, with a score of 1 indicating low decisional conflict and 5 indicating high decisional conflict. All items are based on a 5-point Likert scale ranging from strongly disagree to strongly agree. Total scores and subscale scores can be summed, divided by the number of items, and then converted to a 0 to 100 scale. TPS, on the other hand, measures the degree of interpersonal trust in a physician with regard to dependability, confidence, and confidentiality of information. All items are scored on a 5-point Likert scale ranging from *strongly agree* (5) to *strongly disagree* (1), and a summary measure of trust can be obtained by taking the unweighted mean of the responses (negatively worded items are reverse-scored) and transforming that value to a 0 to 100 scale.
4. *Anxiety*. This will be measured by the 7-item Hospital Anxiety and Depression Scale-Anxiety (HADS-A) [24]. The HADS-A is a self-assessment mood scale scored on a 4-point Likert scale and reported as a sum score ranging from 0 to 21, with a higher score indicating a greater level of anxiety symptoms.
5. *UC disease activity*. This will be measured by the patient-reported 13-item Simple Clinical Colitis Activity Index (SCCAI) [25] and FC. SCCAI is a symptom-based clinical score for UC with a score range of 0 to 19, with a higher score indicating greater disease activity. Although originally intended for clinician use, recent studies have allowed patients to complete the SCCAI themselves [26,27]. FC is an increasingly accepted biomarker for the assessment of disease activity in IBD. The upper limit of the normal range of FC in patients without gut inflammation is well defined as less than 50 µg/g [28].
6. *Clinical outcomes*. These will be captured by 4 items included in the Web-based questionnaire asking patients to report on their use of systemic corticosteroids, need for UC-related surgery, emergency department visits, and hospital admissions. Responses to these items will be used to initiate chart reviews for validation and to calculate: (1) proportion of patients taking steroids, (2) proportion of patients requiring surgery, and (3) number and days of unplanned emergency department visits and hospital admissions.
7. *Work productivity*. This will be measured by the 6-item Work Productivity and Activity Impairment questionnaire (WPAI) [29]. The WPAI measures 4 domains of the impact of disease on impairment in work or other activities (absenteeism, presenteeism, overall work productivity loss, and activity impairment). It is self-reported using a 1-week recall period, and domain scores are expressed as percentage of impairment, with higher scores indicating worse work-related outcomes.
8. *Acceptability of myAID*. This will be measured by a Web-based questionnaire at 2 to 4 weeks from the time of referral (or following the *decision consult* if arranged) for the intervention group. We will seek intervention group participants’ views about the ease of viewing myAID, adequacy of its content, optimal timing for receipt of myAID, and the extent to which it facilitated understanding of their condition and the available treatment options, and ultimately, whether it aided in the discussion of treatment with their clinician. We will also determine whether additional resources were sought or prescribed and whether a *decision consult* was considered necessary in the decision-making process.

Patients who undergo colectomy during the study will complete a modified questionnaire that does not include the SCCAI and will no longer submit stool samples for FC measurement.

### Trial Management

The myAID study group, consisting of 2 gastroenterologists, 1 IBD clinical nurse consultant, 1 psychologist, and 1 research assistant, provides day-to-day oversight of the trial and meets at 1 to 2 weekly intervals to address any queries regarding the patient’s eligibility at the time of referral or logistical issues raised by participating sites. Each study site will have 1 lead clinician to oversee local patient referral activity and act as the liaison for the myAID study group, but sites will not be directly involved in any patient consent, data collection, or follow-up procedures, which will be entirely managed by the myAID study group. The myAID study group will not be involved in any test result interpretation, for example, colonoscopy or FC, treatment decisions, or advice about management, and any such queries from participants will be forwarded directly to the treating team.

### Safety Monitoring and Reporting

As the intervention in this trial does not include drug treatment or procedures, we do not anticipate any safety issues, although we will keep track of the treatment options being selected based on study participants’ reports.

### Withdrawal

Study participants will be able to withdraw from the study whenever they wish without giving a reason and without affecting their care. We will document their reasons for withdrawal if provided as well as any feedback regarding myAID. If a participant misses 1 assessment, we will encourage them to continue the study and complete the subsequent questionnaires and FC testing at the designated time points. Patients who undergo colectomy during the study period will remain in the study unless they specifically choose to withdraw. Data collection from these patients will be altered as previously described.

### Data Management and Record Keeping

The data acquired during the study will be coded and stored as deidentified data on a secure, password-protected computer located at the Ingham Institute for Applied Medical Research and accessed over a secure, virtual private network. Only the members of the myAID study group not directly involved in the participant’s care will have access to identifiable data except for the results of the FC, which will be forwarded directly to the referring clinicians.

### Sample Size and Power

The myAID study design is based on an assumption of 0.5 SD difference between the intervention and control groups for the primary outcome of QoL using the AQoL-8D utility score at 6 months. This estimate fares well with the published study by Gibson et al using AQoL-8D, which identified the scores between those in clinical remission and those with active UC [19]. The sample size has been adjusted for the design effect due to the cluster randomization assuming unequal cluster sizes and can accommodate an intraclass correlation coefficient (ICC) as high as 0.05 to achieve 80% power. Adjusting for this ICC, the estimated required sample size for the full study is 238 patients (119 per group), which will require a minimum of 14 clusters for sufficient recruitment. Assuming 70% of eligible patients consent to study participation and a loss to follow-up rate of 20% at the 6-month end point, we then aim to approach a minimum of 426 patients (213 per group) and recruit 298 patients at baseline.

### Analysis

#### Missing Data

##### General Principles

We will exclude patients for whom only baseline data has been collected. To remain within the primary outcome analysis, patients will need to have completed the Web-based questionnaires across at least three time points (including baseline measures). For each measure, we will summarize the frequency of missing data and assess how this affects the effective sample size and statistical power. If systematic issues are identified, the study statistician and the chief investigator will discuss the findings. Otherwise, appropriate statistical imputation methods will be used to address missing data.

##### Internal Imputation Within a Questionnaire

None of the questionnaires has an official algorithm for imputing individual missing answers. Given the design of the Web-based questionnaire and the results of the pilot study, we anticipate very few patients providing incomplete responses. Any incomplete questionnaires will be immediately flagged on the Web-based platform, and the patient will be contacted to request completion within the designated period. To additionally reduce missing data and make good use of available information, we will impute missing responses within the included measures and will use multiple imputation methods to achieve this depending on the frequency of missing data.

##### External Imputation of Outcome Measures

If the entire questionnaire at a specific time point is missing, we will impute missing scores by appropriate regression models using all available values of that score at other time points for the individual and other participants belonging to the same allocated group. We will also consider using other predictors from patient demographics and disease activity, although they may only play a limited role in predicting missing values.

##### Outcome Data Analysis

The primary analysis will be by *intention to treat* and conducted using multilevel models to account for the correlation of outcomes within a cluster (site). For the primary outcome, the dependent variable will be the AQoL-8D utility score and the exposure variable will be the intervention status (intervention vs control). The unit of analysis will be at the patient level to accommodate the weighting required by unequal cluster (site) sizes. Other independent variables will be added to the models if they are independently associated with AQoL-8D. Multilevel models will also be used to evaluate secondary outcomes.

Multilevel models will further be used to analyze each of the health literacy scores, adjusting for the participant’s age, ethnicity, educational/work status, location (rural vs urban), insurance status, and health status including anxiety and disease activity. All statistical analyses will be performed using SAS 9.4 (SAS Institute).

##### Health Economics Analysis

We will evaluate the additional costs and health outcomes of myAID intervention compared with usual care from the perspectives of the Australian health care system and society within the trial period. This will include all direct costs related to UC, cost of delivering the intervention over 12 months, downstream costs due to selected treatment, hospital visits, and health care utilization and all indirect costs related to the productivity of the participants. Unit costs for health care utilization and medication use will be estimated from chart reviews and linkage to the Medicare Benefits Schedule and the Pharmaceutical Benefits Scheme national databases. Participants’ total health care cost will be the aggregation from the number of services used by the unit cost for the service plus medication costs. All costs will be expressed in 2016 Australian dollars (Aus $) and effects in quality-adjusted life years (QALYs). The AQoL-8D will provide utilities for the estimation of QALYs in the cost-utility analysis. WPAI will provide an estimation of the indirect cost. No discounting will be applied as follow-up is for 12 months only.

### Patient and Public Involvement

We received input from patients and clinicians from a feasibility pilot study, which guided the design of this study and confirmed the suitability of the intervention, as assessed by patients themselves and by clinicians. The research question and outcome measures were influenced by reviewing other clinical studies examining the role of SDM in patients with IBD, including a similar study assessing the use of a DA in CD. Patients were not involved in the decision of the research question or outcome measures. Patients will not be involved in the recruitment of participants or the conduct of the study. We will gather information about the acceptability of the intervention through a Web-based questionnaire, as part of the study. We plan to disseminate the results of the research to study participants and to the rest of the community through national and international conferences and via publications in peer-reviewed journals.

### Ethics and Dissemination

The completed pilot study and this CRCT were approved by the Human Research Ethics Committee of South Western Sydney Local Health District (HREC/15/LPOOL/358) and relevant site-specific ethics committees. We will report the study findings in accordance with the Consolidated Standards of Reporting Trials guidelines, with appropriate acknowledgment and/or authorship for those who have worked on the trial (as per journal authorship guidelines).

## Results

Study recruitment and funding began in October 2016, and recruitment will continue through 2020 at the current projection to ensure adequate numbers are recruited to both arms. Completion of data collection and publication of study results are anticipated in 2021.

## Discussion

With increasing complexity of treatment choices, supporting SDM and effective communication in UC management have become even more important. Studies on other chronic diseases suggest that the use of DAs may be beneficial to both facilitate SDM and improve communication, although there has been only limited experience in UC. Even if available, to implement such a tool on a wider scale, more evidence is required regarding its potential usability, efficacy, and cost-effectiveness.

The results from the myAID study will, therefore, contribute important new evidence to the literature, with comparison of outcomes between patients who routinely access myAID and those who do not, across multiple large IBD centers as well as community-based private practices in Australia. Furthermore, it will provide insight into the decision-making process utilized by Australian patients with UC using specifically designed questionnaires measuring health literacy, empowerment, and quality of decision making. Information gathered from the study will then be used to guide further revisions of the myAID tool and its wider implementation to the rest of Australia and internationally, to support SDM and provide better outcomes for patients with UC.

## Appendix

Multimedia Appendix 1 Reviewers' report - 2015 GESA AbbVie IBD Clinical Research Grant.

## Abbreviations

5-ASA: 5-aminosalicylates
AQoL-8D: Assessment of Quality of Life-8D
CD: Crohn disease
CRCT: cluster randomized controlled trial
DA: decision aid
DCS: Decisional Conflict Scale
FC: fecal calprotectin
HADS-A: Hospital Anxiety and Depression Scale-Anxiety
heiQ: Health Education Impact Questionnaire
HLQ: Health Literacy Questionnaire
IBD: inflammatory bowel disease
ICC: intraclass correlation coefficient
QALYs: quality-adjusted life years
QoL: quality of life
SCCAI: Simple Clinical Colitis Activity Index
SDM: shared decision making
TPS: Trust in Physician Scale
UC: ulcerative colitis
WPAI: work productivity and activity impairment questionnaire
