# Supplementary material for: A Web-Based Decision Aid (myAID) to Enhance Quality of Life, Empowerment, Decision Making, and Disease Control for Patients With Ulcerative Colitis: Protocol for a Cluster Randomized Controlled Trial
Source: JMIR Res Protoc. 2020 Jul 10;9(7):e15994. doi: 10.2196/15994 (PMC7382012; doi:10.2196/15994)
Supplement: Multimedia Appendix 1 [file resprot_v9i7e15994_app1.pdf]

2015 GESA ABBVIE IBD CLINICAL RESEARCH GRANT

2015 GESA Abbvie IBD Clinical Research Grant

The GESA AbbVie IBD Research Grant aims to support important innovative clinical and scientific research that will ultimately benefit patients with IBD.

Eligibility:

- Be an Australian citizen or hold Permanent Australian Residency.
- Be a financial member of GESA for at least one year prior to application for this fellowship.
- Be an established investigator as evidenced by previous or present project or program grant funding (ie. From NHMRC, ARC, NIH, hospital foundation or university sources)

Assessment Criteria:

- Please rank the applicants in order of merit on the basis of a maximum of 100 points, and whenever possible avoid giving equal ranking.
- Indicate the level beyond which you feel support is not warranted in your ranking.
- Please use the following points system.

**A**	**Clinical Significance and Innovation-**Assess the potential of the proposal to increase knowledge about human health, diagnosis, biology of IBD, or the application of new ideas, procedures or technologies related to IBD	Maximum Points<br>25
**B**	**Feasibility & Experimental Design-**Assess the strengths and weaknesses of the research plan and the experimental design. In addition, evaluate the feasibility of the proposed research i.e. does the research plan adequately address the stated hypothesis and objectives and do the applicants have the skills and techniques established to successfully complete the proposal	Maximum Points<br>50
**C**	**Track Record Relative to Opportunity-**Grant success, translation of Grant funding into publications, number, quality and impact of publications, invitations to speak at significant conferences, and involvement in peer review and the discipline	25

2015 GESA ABBVIE IBD CLINICAL RESEARCH GRANT

Reviewers:__________GESA Research Committee Awards Panel___________

Reviewer No.	APPLICANTS	A: Out of 25	B: Out of 50	C Out of 25	Total: Out of 100	Rank	Comments
1.	CONNOR, Susan	22	40	20	82	1	no comment
2.	CONNOR, Susan	10	30	10	50	5	no comment
3.	CONNOR, Susan	21	34	20	75	2	no comment
4.	CONNOR, Susan	23	42	22	87	1	"An all-of-person caring approach"
5.	CONNOR, Susan	20	38	20	78	3	no comment
6.	CONNOR, Susan	20	25	20	65	2	"Thorough application, innovative aspects, good team, good track record of other CIs"
